# Comparison study of the axial length measured using the new swept-source optical coherence tomography ANTERION and the partial coherence interferometry IOL Master

**DOI:** 10.1371/journal.pone.0244590

**Published:** 2020-12-31

**Authors:** Kook Young Kim, Gon Soo Choi, Min Seok Kang, Ungsoo Samuel Kim

**Affiliations:** Kim's Eye Hospital, Seoul, Korea; Nicolaus Copernicus University, POLAND

## Abstract

**Purpose:**

To compare a biometer using swept-source optical coherence tomography (SS-OCT) with a partial coherence interferometry (PCI)-based biometer in measurements of two ocular biometry parameters, i.e., the axial length and anterior cornea curvature.

**Methods:**

We compared the two biometers SS-OCT (ANTERION, Heidelberg Engineering Inc., Heidelberg, Germany) and PCI (IOL Master, Carl Zeiss Meditec, Jena, Germany) in terms of the axial length (AL) and corneal curvature (K) measurements of 175 eyes. Paired *t*-tests were used to compare the two biometers. Agreement between the biometers was evaluated using the Bland–Altman method.

**Results:**

The mean age was 36.0 ± 25.6 years (range: 5 to 85 years). The mean axial length was 24.42 ± 0.13 mm for SS-OCT and 24.45 ± 0.14 mm for PCI. The mean corneal curvature was significantly different between the two biometry in flat K (K1) but not in steep K (K2). The limit of agreement was -0.15 to 0.21 in the axial length, -1.18 to 0.83 in K1, and -1.06 to 0.95 in K2. All above ocular biometric measurements between SS-OCT and PCI correlated significantly (Pearson's correlation, p<0.001).

**Conclusions:**

The axial length measured using SS-OCT is useful in clinical practice. It shows a good correlation and agreement with that measured using PCI. However, the axial length and corneal curvature measured using SS-OCT cannot be used interchangeably with that measured using PCI in clinical practice.

## Introduction

The precise measurement of the axial length is important in the field of ophthalmology owing to various applications. For example, it is the fundamental index to select the correct intraocular lens or follow the progression of myopia. To calculate the axial length, the ultrasonographic A-scan is the traditional method, and partial coherence interferometry (PCI), such as IOLMaster500 (Carl Zeiss Meditec, Jena, Germany), is also a popular technique. This device measures the axial length by calculating the time difference between the reflected rays using 780-nm infrared beam of short-coherence light with only a single refractive index, although the refractive index of each tissue is different. Corneal curvature is measured among six hexagonal reflected lights on a 2.3mm radius on the cornea. PCI yields a precise resolution of 0.01 mm [1] and an error less than ± 0.05 mm in repeated measurements, making it more advantageous to detect changes in the fine lining [2]. Moreover, because it employs non-contact biometry, it is not significantly affected by the proficiency of the examiner, and its accuracy and reproducibility are superior to those of the A-scan [3].

Recently, high-resolution swept-source optical coherence tomography (SS-OCT) has been introduced to analyze, not only the posterior segments, but also the anterior segment of the eyeball. It can demonstrate the structure of the eyeball, corneal topography, and biometry, including the axial length, owing to the greater tissue penetration depth by the light source. SS-OCT devices currently used in clinical practice are Argos (Movu, Santa Clara, CA), IOLMaster 700 (Carl Zeiss Meditec, Jena, Germany), and OA-2000 (Tomey, Nagoya, Japan). It is well known how accurate are these clinical SS-OCT measurements in comparison with the previously described IOL Master 500 [4–6]. The Anterion (Heidelberg Engineering Inc., Heidelberg, Germany) is a new high-resolution SS-OCT device capable of capturing a wider scan depth (14.5 mm) and scan width (16.5 mm), with a light source of 1300-nm wavelength, compared to the existing SS-OCT devices, and of measuring the axial length in the range of 14–32 mm.

There have been no studies comparing the reliability and agreement of this new SS-OCT device with the conventional PCI biometer. Therefore, the objective of the present study was to compare the ANTERION (SS-OCT) and IOLMaster 500 (PCI) in measurements of the major ocular biometry parameters: the axial length, anterior chamber depth (ACD), and anterior cornea curvature.

## Material and methods

We retrospectively reviewed the medical records of patients, who had been undergone SS-OCT and PCI at Kim's Eye Hospital from February 2020 to June 2020 for the analysis of their ocular biometry. The study protocol was approved by the Institutional Review Board (IRB number: KEH 2020-07-003-003) at Kim's Eye Hospital, Seoul, Korea, and the study was conducted in accordance with the tenets of the Declaration of Helsinki.

In this study, we enrolled a total of 175 eyes of 107 patients, including 94 eyes of men and 81 eyes of women, with a mean age of 36.0 ± 25.6 years (range: 5 to 85 years). Among the participants, the total number of cataract patients was 64; all patients with mild cataract had a best-corrected visual acuity (BCVA) of 0.7(decimal value) or more; and no moderate to severe nuclear sclerosis affecting the refractive error. All other patients, aged 40 years or younger, had a BCVA of 1.0 without ocular abnormalities. The patients with previous ocular trauma, those who had been undergone prior refractive surgery, and those with corneal opacity, or another disease that affects visual acuity except cataract were excluded.

### Instruments

All tests were conducted by one skilled examiner in a dark room with SS-OCT and PCI (version 5.40, manufacture). The order of examination of participants was selected randomly. SS-OCT measurements of the axial length, anterior chamber depth and corneal curvature were performed using the cataract application mode. The axial length was defined as the distance between the anterior corneal tear film and the retinal pigment epithelium (RPE) along the line of sight. The axial length measurement was calculated on three subsets of data. The algorithm checks how many measurements are within 0.05 mm of each other. If all three subsets measurements are within 50 μm, the mean and standard deviation for axial length is calculated. For non-pathological eyes with a clearly defined RPE peak, the standard deviation of the axial length should be less than 0.02 mm. The anterior chamber depth is defined as the distance from the anterior corneal surface to the anterior lens surface, measured perpendicular to the anterior corneal surface and along the visual axis. The corneal curvature was measured in a total of 65 radial B-scan images (256 A-scans per B-scan) and acquired in less than 1 s. The simulated anterior curvature was analyzed in the 3-mm zone of the central cornea.

Using PCI, the axial length was measured and optical A-scans were obtained along the visual axis [7]. The anterior chamber depth is measured along the visual axis from the corneal epithelium to the anterior surface of lens. The PCI takes five simultaneous anterior chamber depth measurements and calculate the mean value. The anterior corneal curvature was also obtained from six hexagonal arrays reflected from the central cornea face in a plane approximately 2.3 mm in diameter. The device records the reflection of these images measuring the separation of the opposite pairs of light spots and calculating the corneal radii and toroidal surface curvature.

### Statistical analysis

Data were analyzed using SPSS version 24.0 (IBM Corporation, Armonk, NY, USA), and Medcalc software (version 18.2.1 Mariakerke, Belgium). The Shapiro–Wilk test was used to evaluate the normality of numerical data. A paired *t*-test was used to compare the axial lengths, anterior chamber depth and corneal curvature between two biometers. Pearson's product-moment correlation coefficient was used to analyze the correlation between parameters of two devices. The Bland–Altman limits-of-agreement (LoA) [8] was used to evaluate the agreement between both biometers for the axial length, ACD and anterior corneal curvature values. Corneal astigmatism was additionally analyzed by vector analysis to find out the changes in cylinder power and cylinder axis as previous study [9]. Statistical significance was set at *p* < 0.05.

## Results

**Fig 1:**
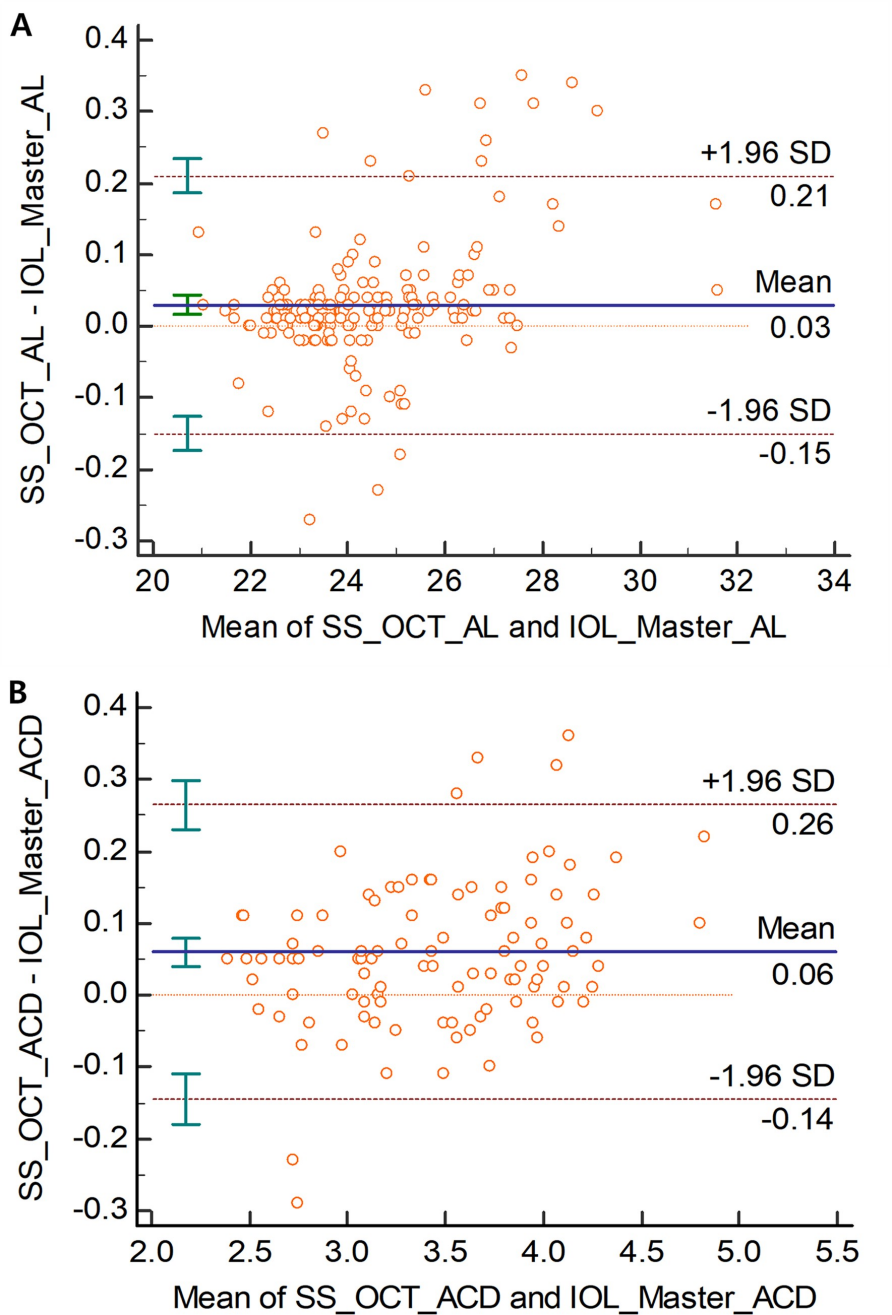
Bland–Altman plots of agreement between swept-source optical coherence tomography and partial coherence interferometry. (A) axial length (AL) for 175 eyes, (B) anterior chamber depth (ACD) for 104 eyes. The solid line shows the mean difference (bias). Upper and lower dashed lines imply 95% limits of agreement.

The mean spherical equivalent was -2.28 ± 0.25 D (range: -17.25 to +3.5D), and BCVA was 0.98 ± 0.00 (decimal value). The mean axial length were 24.42 ± 0.13 mm (range: 21.01 to 31.65 mm) for SS-OCT and 24.45 ± 0.14 mm for PCI (range: 20.88 to 31.57 mm) in 175 eyes. The mean difference was -0.03 ± 0.09 with a significant difference in the paired *t*-test (*p*<0.001). The axial length LoA was -0.15 to 0.21. The mean ACD were 3.50 ± 0.57 mm (range: 3.10 to 4.93 mm) for SS-OCT in 175 eyes and 3.44 ± 0.54 mm for PCI (range: 3.07 to 4.75 mm) in 104 eyes. The mean difference was 0.06 ± 0.10 with a significant difference in the paired *t*-test (*p*<0.001). The LoA of ACD was -0.14 to 0.26 (Table 1) (Fig 1).

**Table 1:** Mean value, mean difference and 95% limit of agreement of axial length in 175 eyes and anterior chamber depth in 104 eyes readings of the two biometry devices.

		Mean value ± SD (mm)	Mean difference ± SD (95% confidence interval) (mm)	p-value*	95% LoA
**Axial length**	PCI	24.45 ± 0.14	-0.03 ± 0.09 (-0.04, -0.02)	<0.001	0.36 (-0.15 to 0.21)
	SS-OCT	24.42 ± 0.13			
**ACD**	PCI	3.44 ± 0.54	0.06 ±0.10 (0.04, 0.08)	<0.001	0.40 (-0.14 to 0.26)
	SS-OCT	3.50 ± 0.57			

*: p-value of paired T-test between two devices.

SD: standard deviation; LoA: limits of agreement; PCI: partial coherence interferometry, SS-OCT: swept-source optical coherence tomography; ACD: anterior chamber depth.

**Fig 2:**
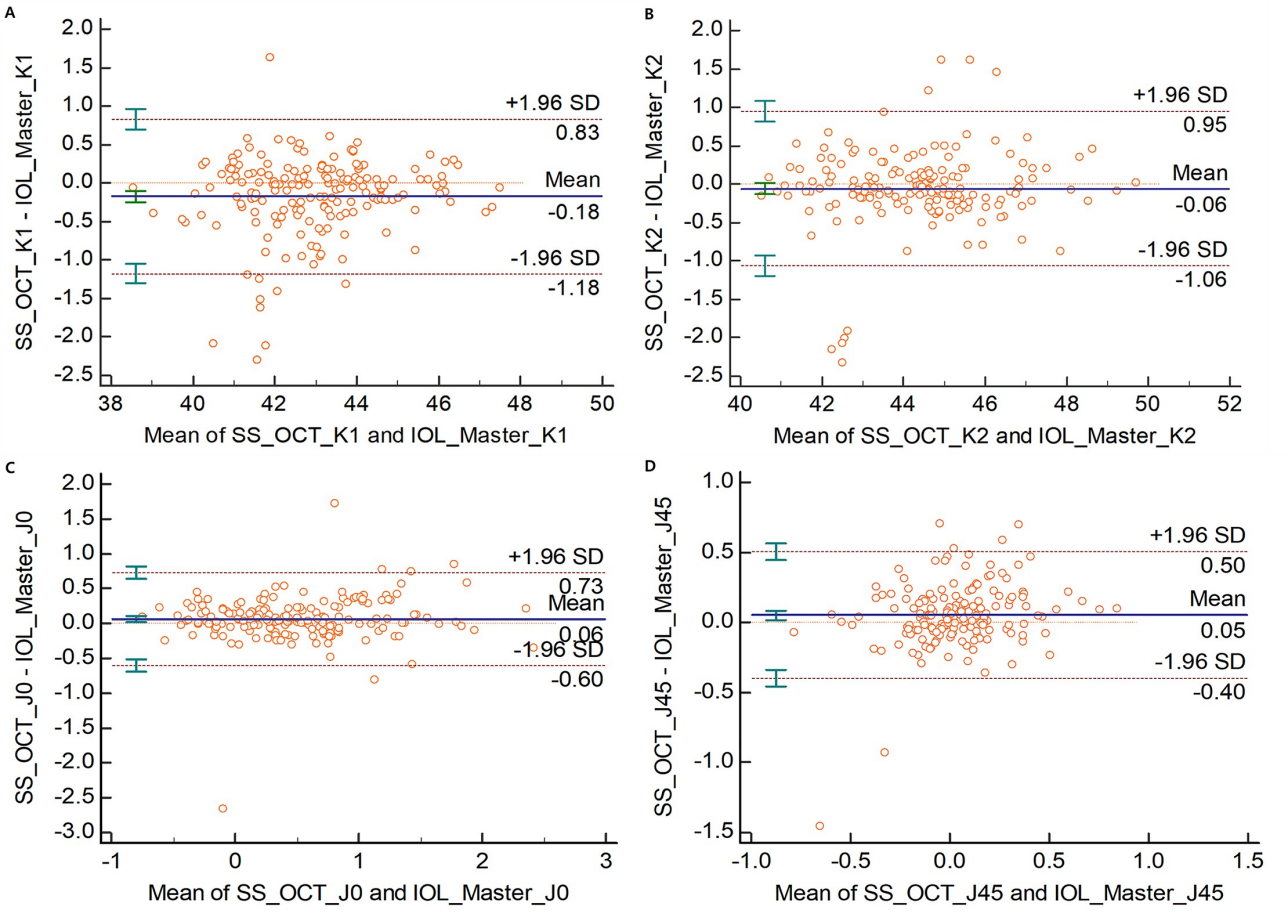
Bland–Altman plots of agreement in the evaluation of the corneal curvature. (A) flat K (K1), (B) steep K (K2), (C) J0, (D) J45. The solid line shows the mean difference (bias). Upper and lower dashed lines imply 95% limits of agreement.

**Fig 3:**
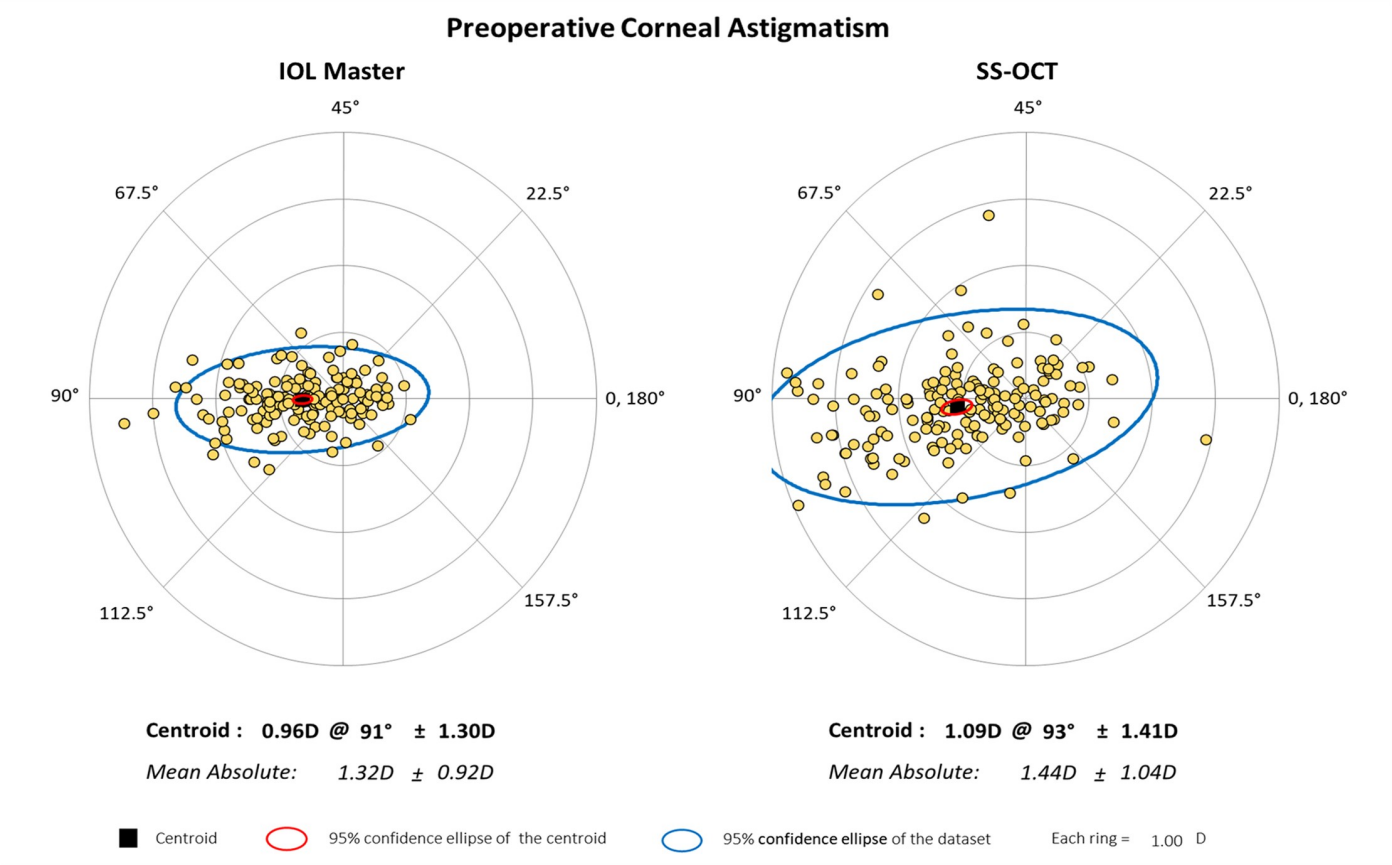
Preoperative astigmatism using double-angle plot of two biometry.

The mean corneal curvature was as follows: SS-OCT, K1: 42.89 ± 1.71 D (range: 38.51 to 47.47), K2: 44.33 ± 1.83 D (range: 40.45 to 49.72); PCI, K1: 43.06 ± 1.66 D (range: 38.57 to 47.54), K2: 44.38 ± 1.78 D (range: 40.61 to 49.71). There was statistically significant difference between the two groups in flat K (K1) but not in steep K(K2). The mean corneal curvature difference was 0.18 ± 0.51 D in K1 and 0.06 ± 0.51 D in K2, and the LoA was -1.18 to 0.83 in K1 and -1.06 to 0.95 in K2. The mean J0 value was 0.48 ± 0.60 D (range: -0.44 to 2.59) in PCI and 0.54 ± 0.64 D (range: -0.44 to 0.79) in SS-OCT, with a mean difference of -0.06 ± 0.03 (p-value = 0.014, LOA was -0.60 to 0.73). The mean J45 value was 0.01 ± 0.24 D (range: -0.68 to 2.46) in PCI and 0.06 ± 0.30 D (range: -0.15 to 0.89) in SS-OCT, with a mean difference of -0.05 ± 0.02 (p-value = 0.004, LOA was -0.40 to 0.50) (Table 2) (Fig 2). The preoperative astigmatism was represented with a double angle plot tool according to previous study (Fig 3) [10].

**Table 2:** Mean value, mean difference, and 95% limit of agreement (LoA) of keratometric values (K1, K2, J0 and J45) between two biometry.

		Mean value ± SD (D)	Mean difference ± SD (95% confidence interval) (D)	p-value*	95% LoA
**K1**	PCI	43.06 ± 1.66	0.18 ± 0.51 (0.10, 0.25)	<0.001	2.01 (-1.18 to 0.83)
	SS-OCT	42.89 ± 1.71			
**K2**	PCI	44.38 ± 1.78	0.06 ± 0.51 (-0.02, 0.13)	0.136	2.01 (-1.06 to 0.95)
	SS-OCT	44.33 ± 1.83			
**J0**	PCI	0.48 ± 0.60	-0.06 ± 0.03 (-0.11, -0.01)	0.014	1.33 (-0.60 to 0.73)
	SS-OCT	0.54 ± 0.64			
**J45**	PCI	0.01 ± 0.24	-0.05 ± 0.02 (-0.09, -0.02)	0.004	0.90 (-0.40 to 0.50)
	SS-OCT	0.06 ± 0.30			

*: p-value of paired T-test between two devices.

SD: standard deviation; D: diopter; LoA: limits of agreement; K1: flat keratometry; K2 steep keratometry; PCI: partial coherence interferometry, SS-OCT: swept-source optical coherence tomography.

All above ocular biometric measurements between SS-OCT and PCI correlated significantly (Pearson's correlation, *p*<0.001) (Table 3).

**Table 3:** Pearson correlation of ocular parameters reading between two biometry.

	Axial length	ACD	K1	K2	J0	J45
**Correlation coefficient**	0.999	0.984	0.955	0.960	0.854	0.652
**P-value**	<0.001	<0.001	<0.001	<0.001	<0.001	<0.001

ACD: anterior chamber depth; K1: flat keratometry; K2 steep keratometry.

## Discussion

Fișuș AD et al. showed that The ANTERION had a good correlation and agreement with IOLMaster 700 for all the biometric parameters including axial length, keratometry, anterior chamber depth, lens thickness and central corneal thickness [11]. This is the first study in the literature in which ANTERION SS-OCT was compared with the previous considered gold standard PCI -IOLMaster 500 for the the biometric parameter measurements. Ramón RM et al. showed that ANTERION was a SS-OCT device with good repeatability for different ocular biometric measurements [12].

SS-OCT using the 1300-nm wavelength has garnered much attention for use in ophthalmic imaging owing to its deeper penetration in tissue than 780-nm light sources. The use of longer wavelengths of SS-OCT allows for a higher permissive power intensity on the eye than it is not possible with shorter wavelengths and the loss of optical signal is smaller due to less dispersion. The anterior segment of the eye measured by SS-OCT is well imaged due to less optical signal loss and high permissive power in the eye; with a good axial length precision as PCI due to RPE reflects very well longer wavelengths. It is important to measure the axial length accurately because the accuracy of the axial length measurement is responsible for more than half of the causes of refractive errors after cataract surgery [13, 14].

Axial length is a key ocular biometric parameter in IOL power calculation. In this study, the measured mean difference in axial length was -0.03 ± 0.09 mm, the axial length measurement of PCI was slightly longer. There was a significant statistical difference in the axial length between the two biometry. Several studies have compared different commercial SS-OCT as IOL Master 700, ARGOS or OA-2000, with IOL Master 500; however, there have been controversies on the correlation between SS-OCT and PCI. An Y et al. expected significant differences in the measured values of the axial length between ARGOS SS-OCT and IOLMaster 500 due to different measurement principles, but there was no statistical significance in patients who were undergone to cataract surgery (ARGOS: 24.56 ± 2.16 mm; IOLMaster 500: 24.58 ± 2.22 mm, *p*>0.05) [4]. Another previous study comparing ARGOS and IOLMaster 500 showed no significant difference in the axial length measurement [15]. Yang JY et al. found that between IOLMaster 700 (SS-OCT) and 500 in the group of high-myopia patients, the axial length measured by IOLMaster 700 was statistically significantly longer (IOLMaster 700: 27.11 ± 3.00 mm; IOLMaster 500: 27.05 ± 2.99 mm, *p*<0.001) [5]. Du Y-L et al. showed that there was a statistically significant difference between OA-2000 (SS-OCT, 29.08 ± 2.31 mm) and IOLMaster 500 (29.06 ± 2.30mm) [6]. This discrepancy in the axial length between two methods is due to the basic principles of the axial length measurement. IOLMaster uses the refractive index (1.3549) to calculate the axial length, while the SS–OCT biometer uses different refractive indices depending on the ocular media. For example, ARGOS SS-OCT measures the axial length by applying the following refractive index: 1.376 for the cornea, 1.336 for the aqueous and vitreous humours; and 1.410 for the lens [16]. In general, conclusions from past studies were that there were no clinically significant differences in the actual mean values of the axial length and that there was a high correlation between the two models. Two SS-OCT (IOLMaster 700 and ANTERION) were compared by Fisus AD et al. This study showed that the mean axial length was 23.55 ± 1.18 mm (range: 20.09to 28.99) of IOLMaster 700 and 23.54 ± 1.18 mm (range: 20.10to 29.19) of ANTERION and the mean arithmetic difference between devices was 0.01 ± 0.03 mm, statistically significant (p<0.001) [11]. In the comparative studies on the axial length of many different biometry, some studies have statistical differences and others do not. In common, there was no difference that could cause a difference in the clinically meaningful IOL calculation value. These differences between two biometry devices would not be clinically significant because a 0.00~0.05-mm difference in the axial length would result in a less than 0.1-D difference in postoperative refractive errors [17]. In addition, the agreement between SS-OCT and PCI was also clinically good (range of 95% LoA, 0.36, -0.15 to 0.21), and their correlation was significantly good in this study. Therefore, it is assumed that the axial length measured by SS-OCT could be used clinically without much difference compared to the conventional PCI.

This study included Asian patients who were undergone to ophthalmic examinations, had a myopic refractive error (-2.28 ± 0.25 D) [18]. Further research will require more subjects with various refractive errors. As subjects of various refractive errors and various ages are included in the study, we think that the actual clinical situation can be more reflected.

Although this study was retrospective in nature, it is meaningful, as it is the first study to compare the measurement of the axial length by SS-OCT among patients of various ages. Forty-five eyes of patients under the age of 10 years included in this study were also measured at a faster rate than with conventional PCI, enabling an accurate measurement of the axial length.

In this study, the PCI could not measure ACD in some cases, so only 104 eyes measured by both instruments were compared and analyzed. The mean ACD (central cornea thickness + anterior aqueous depth) of ANTERION was 3.20 ± 0.42 mm (range: 1.97 to 4.33 mm), there were significant difference capered with IOLMaster700 (3.13 ± 0.43 mm, range: 1.89 to 4.28 mm) [11]. As the viewer (HEYEX, version 2.5; Heidelberg Engineering) of the ANTERION is upgraded, the ACD and anterior aqueous depth (AQD; defined as the distance from the apex of the anterior lens surface to the apex of the corneal endothelium) are expressed separately. The previous study is a result of adding CCT to AQD for comparison with the IOLMaster 700, and there may be a difference from this study. The comparative study with SS-OCT CASIA1000 showed the AQD were 2.83 ± 0.53 mm (CASIA1000) and 2.89 ± 0.70 mm (ANTERION) with no significant difference (p value = 0.06) [19]. IOLMaster500 use the principle of PCI for axial length measurement, but the anterior chamber depth is measured by optical principles using a non-PCI method. The ACD was measured by a 0.7 mm-wide slit beam of light which is directed at a 30-degree angle into the anterior chamber and calculated the distance between light reflections on the anterior corneal surface and the anterior lens surface. SS-OCT is measured by autosegmentation of ACD and AQD by directly tomographic image of the cornea and lens based on the optical axis. We thought that this difference in the measurement principle may cause a difference in the ACD value. Hoffer et al. compared IOLMaster 700 and a LENSTAR for mean difference of ACD was 0.02 mm, which, although statistically significant, is not clinically relevant [20]. This difference in ACD can be a factor of error in IOL calculations using effective lens position (ELP) as a major variable, such as Haigis formula, and may cause a change in refractive power after cataract surgery in shallow ACD or high myopia [21].

The difference in the flat and steep keratometric value is thought to be due to differences in measurement methods. PCI uses a distance-independent telecentric keratometry system, which measures the curvature of the light source by projecting it to the cornea [22]. IOLMaster 500 measures the corneal curvature at six hexagonal points in a central 2.3 mm area, and SS-OCT measures the simulated anterior corneal curvature in a 3mm zone with a 65-radial scan, which is thought to make a difference in the average corneal curvature measurement. It is thought that ANTERION SS-OCT measures flatter than IOL Master 500 because it measures a larger number of B-scans in a wide range.

Astigmatism should be expressed in the axis and cylindric power, and the sphero-cylinder notation used in clinical practice is effective in representing the axis and cylindric power. However, if the axis does not coincide, it becomes difficult to calculate the refraction power. In order to compensate for this problem, we analyzed the astigmatism by converting it to the power vector method and there was good agreement between two biometry. Fig 3 which represented with a double angle plot tool of astigmatism, showed 95% limit of agreement of the centroid (blue ellipse) was larger than PCI, that mean the higher the variability. This is thought to occur because SS-OCT measures a relatively large number of scans and a wide range of astigmatism.

There are some limitations to this study. First, Although the differences and agreement of the biometric parameters between two different biometry were analyzed, but it was not shown whether these differences were related to errors in actual clinical trials such as refraction errors occurring during IOL calculation. Therefore, there is a need for further research related to the above limitation. Second, although the total number of study subjects was not small, factors affecting the difference and error in measured values could not be completely excluded because subjects with too various ages and refractive indexes were studied. Further studies are required to measure and compare many subjects by age group or specific refractive error group. However, in clinical practice, the measurement of the axial length in clinics is not limited to a specific group or specific refractive error of subjects, so results of this study may reflect relatively more of the actual clinical situation. In addition, selection bias may occur because the study was not conducted with only one eye of the subject, but partially included both eyes. But each eye was judged as an independent entity that did not affect each other on the test result, and the study was conducted.

In conclusion, this study showed a good correlation and agreement so that ANTERION SS-OCT could replace the existing PCI in measuring the axial length, ACD and keratometric value. There was no clinically relevant difference between the measured values to cause a significant error in clinical application, but there was a statistically significant difference. Although differences were found to be small, the parameters measured by two biometry should not be used interchangeably.

## Supporting information

S1 File Data analyzed. (XLSX)

## References

[1] Verhulst E, Vrijghem JC. Accuracy of intraocular lens power calculations using the Zeiss IOL master. A prospective study. Bulletin de la Societe belge d'ophtalmologie. 2001;(281):61–5. Epub 2001/11/13. . 11702645

[2] Kimura S, Hasebe S, Miyata M, Hamasaki I, Ohtsuki H. Axial length measurement using partial coherence interferometry in myopic children: repeatability of the measurement and comparison with refractive components. Japanese journal of ophthalmology. 2007;51(2):105–10. 10.1007/s10384-006-0410-5 17401619

[3] Connors R III, Boseman P III, Olson RJ. Accuracy and reproducibility of biometry using partial coherence interferometry. Journal of Cataract & Refractive Surgery. 2002;28(2):235–8. 11821202 10.1016/s0886-3350(01)01179-8

[4] An Y, Kang E-K, Kim H, Kang M-J, Byun Y-S, Joo C-K. Accuracy of swept-source optical coherence tomography based biometry for intraocular lens power calculation: a retrospective cross–sectional study. BMC ophthalmology. 2019;19(1):30 10.1186/s12886-019-1036-y 30678658 PMC6346505

[5] Yang JY, Kim HK, Kim SS. Axial length measurements: Comparison of a new swept-source optical coherence tomography–based biometer and partial coherence interferometry in myopia. Journal of Cataract & Refractive Surgery. 2017;43(3):328–32. 10.1016/j.jcrs.2016.12.023 28410713

[6] Du Y-L, Wang G, Huang H-C, Lin L-Y, Jin C, Liu L-F, et al Comparison of OA-2000 and IOL Master 500 using in cataract patients with high myopia. International journal of ophthalmology. 2019;12(5):844 10.18240/ijo.2019.05.23 31131247 PMC6520281

[7] Santodomingo-Rubido J, Mallen E, Gilmartin B, Wolffsohn J. A new non-contact optical device for ocular biometry. British Journal of Ophthalmology. 2002;86(4):458–62. 10.1136/bjo.86.4.458 11914218 PMC1771084

[8] Bland JM, Altman DG. Comparing methods of measurement: why plotting difference against standard method is misleading. The lancet. 1995;346(8982):1085–7. 10.1016/s0140-6736(95)91748-9 7564793

[9] Thibos LN, Horner D. Power vector analysis of the optical outcome of refractive surgery. Journal of Cataract & Refractive Surgery. 2001;27(1):80–5. 10.1016/s0886-3350(00)00797-5 11165859

[10] Abulafia A, Koch DD, Holladay JT, Wang L, Hill W. Pursuing perfection in intraocular lens calculations: IV. Rethinking astigmatism analysis for intraocular lens-based surgery: Suggested terminology, analysis, and standards for outcome reports. Journal of Cataract & Refractive Surgery. 2018;44(10):1169–74. 30243391 10.1016/j.jcrs.2018.07.027

[11] Fisus AD, Hirnschall ND, Findl O. Comparison of two swept-source optical coherence tomography-based biometry devices. Journal of Cataract & Refractive Surgery. 2020 10.1097/j.jcrs.0000000000000373 32769752

[12] Ruíz-Mesa R, Aguilar-Córcoles S, Montés-Micó R, Tañá-Rivero P. Ocular biometric repeatability using a new high-resolution swept-source optical coherence tomographer. Expert Review of Medical Devices. 2020;(just-accepted). 10.1080/17434440.2020.1772050 32425075

[13] Olsen T. Sources of error in intraocular lens power calculation. Journal of Cataract & Refractive Surgery. 1992;18(2):125–9. 10.1016/s0886-3350(13)80917-0 1564648

[14] Norrby S. Sources of error in intraocular lens power calculation. Journal of Cataract & Refractive Surgery. 2008;34(3):368–76. 10.1016/j.jcrs.2007.10.031 18299059

[15] Higashiyama T, Mori H, Nakajima F, Ohji M. Comparison of a new biometer using swept-source optical coherence tomography and a conventional biometer using partial coherence interferometry. Plos one. 2018;13(4):e0196401 10.1371/journal.pone.0196401 29689110 PMC5918161

[16] Shammas HJ, Ortiz S, Shammas MC, Kim SH, Chong C. Biometry measurements using a new large-coherence–length swept-source optical coherence tomographer. Journal of Cataract & Refractive Surgery. 2016;42(1):50–61. 10.1016/j.jcrs.2015.07.042 26948778

[17] Eibschitz-Tsimhoni M, Tsimhoni O, Archer SM, Del Monte MA. Effect of axial length and keratometry measurement error on intraocular lens implant power prediction formulas in pediatric patients. Journal of American Association for Pediatric Ophthalmology and Strabismus. 2008;12(2):173–6. 10.1016/j.jaapos.2007.10.012 18423341

[18] Holden BA, Fricke TR, Wilson DA, Jong M, Naidoo KS, Sankaridurg P, et al Global prevalence of myopia and high myopia and temporal trends from 2000 through 2050. Ophthalmology. 2016;123(5):1036–42. 10.1016/j.ophtha.2016.01.006 26875007

[19] Pardeshi AA, Song AE, Lazkani N, Xie X, Huang A, Xu BY. Intradevice repeatability and interdevice agreement of ocular biometric measurements: A comparison of two swept-source anterior segment OCT devices. Translational Vision Science & Technology. 2020;9(9):14–. 10.1167/tvst.9.9.14 32879770 PMC7442878

[20] Hoffer KJ, Hoffmann PC, Savini G. Comparison of a new optical biometer using swept-source optical coherence tomography and a biometer using optical low-coherence reflectometry. Journal of Cataract & Refractive Surgery. 2016;42(8):1165–72. 10.1016/j.jcrs.2016.07.013 27531293

[21] Ning X, Yang Y, Yan H, Zhang J. Anterior chamber depth—a predictor of refractive outcomes after age-related cataract surgery. BMC ophthalmology. 2019;19(1):134 10.1186/s12886-019-1144-8 31238910 PMC6591866

[22] Karunaratne N. Comparison of the P entacam equivalent keratometry reading and IOL M aster keratometry measurement in intraocular lens power calculations. Clinical & experimental ophthalmology. 2013;41(9):825–34. 23601493 10.1111/ceo.12124

